# Identification of entomopathogenic nematodes and symbiotic bacteria from Nam Nao National Park in Thailand and larvicidal activity of symbiotic bacteria against *Aedes aegypti* and *Aedes albopictus*

**DOI:** 10.1371/journal.pone.0195681

**Published:** 2018-04-11

**Authors:** Temsiri Yooyangket, Paramaporn Muangpat, Raxsina Polseela, Sarunporn Tandhavanant, Aunchalee Thanwisai, Apichat Vitta

**Affiliations:** 1 Department of Microbiology and Parasitology, Faculty of Medical Science, Naresuan University, Phitsanulok, Thailand; 2 Centre of Excellence in Medical Biotechnology (CEMB), Faculty of Medical Science, Naresuan University, Phitsanulok, Thailand; 3 Department of Microbiology and Immunology, Faculty of Tropical Medicine, Mahidol University, Bangkok, Thailand; Universita degli Studi di Camerino, ITALY

## Abstract

Entomopathogenic nematodes (EPNs) that are symbiotically associated with *Xenorhabdus* and *Photorhabdus* bacteria can kill target insects via direct infection and toxin action. There are limited reports identifying such organisms in the National Park of Thailand. Therefore, the objectives of this study were to identify EPNs and symbiotic bacteria from Nam Nao National Park, Phetchabun Province, Thailand and to evaluate the larvicidal activity of bacteria against *Aedes aegypti* and *Ae*. *albopictus*. A total of 12 EPN isolates belonging to *Steinernema* and *Heterorhabditis* were obtained form 940 soil samples between February 2014 and July 2016. EPNs were molecularly identified as *S*. *websteri* (10 isolates) and *H*. *baujardi* (2 isolates). Symbiotic bacteria were isolated from EPNs and molecularly identified as *P*. *luminescens* subsp. *akhurstii* (13 isolates), *X*. *stockiae* (11 isolates), *X*. *vietnamensis* (2 isolates) and *X*. *japonica* (1 isolate). For the bioassay, bacterial suspensions were evaluated for toxicity against third to early fourth instar larvae of *Aedes* spp. The larvae of both *Aedes* species were orally susceptible to symbiotic bacteria. The highest larval mortality of *Ae*. *aegypti* was 99% after exposure to *X*. *stockiae* (bNN112.3_TH) at 96 h, and the highest mortality of *Ae*. *albopictus* was 98% after exposure to *P*. *luminescens* subsp. *akhurstii* (bNN121.4_TH) at 96 h. In contrast to the control groups (*Escherichia coli* and distilled water), the mortality rate of both mosquito larvae ranged between 0 and 7% at 72 h. Here, we report the first observation of *X*. *vietnamensis* in Thailand. Additionally, we report the first observation of *P*. *luminescens* subsp. *akhurstii* associated with *H*. *baujardi* in Thailand. *X*. *stockiae* has potential to be a biocontrol agent for mosquitoes. This investigation provides a survey of the basic diversity of EPNs and symbiotic bacteria in the National Park of Thailand, and it is a bacterial resource for further studies of bioactive compounds.

## Introduction

*Xenorhabdus* and *Photorhabdus* are Gram-negative bacteria of the family Enterobacteriaceae and are symbiotically associated with the entomopathogenic nematodes (EPNs), *Steinernema* and *Heterorhabditis* respectively [1]. The infective juvenile stages (IJs) of EPNs containing symbiotic bacteria in their midguts live in the soil of diverse ecological systems [2]. The nematodes can kill the target insects via direct infection through the promotion of the secondary metabolites and toxins produced by symbiotic bacteria. When the IJs of nematodes enter the mouth, anus or spiracles of insect hosts, symbiotic bacteria are released from their intestines into the hemocoel of target insects [3]. Subsequently, the symbiotic bacteria produce several toxins or secondary metabolites, killing the insects by induction of immunosuppression and pervasion of the hemolymph [4]. Finally, the symbiotic bacteria replicate rapidly and cause septicemia in insects [5]. In addition, several secondary metabolites produced by the symbiotic bacteria have been reported as bioactive compounds with activities including cytotoxic, antimicrobial, antiparasitic and insecticidal ones [6, 7].

Earlier studies on the larvicidal activity of symbiotic bacteria have demonstrated that *Photorhabdus* insect-related (Pir) protein had a high toxicity against *Aedes aegypti* and *Aedes albopictus*, a main vector of Dengue virus [8]. In addition, cell suspensions of *P*. *luminescens* and *X*. *nematophila* were orally toxic to *Ae*. *aegypti* [9]. The mortality rate of *Ae*. *aegypti* was further increased when the Cry4Ba protein from *Bacillus thuringiensis* and suspensions of *P*. *luminescens* and *X*. *nematophila* were mixed [10]. The mortality of *Ae*. *aegypti* larvae was 20% when fed with Cry4Ba (5 ηg/ml) alone. At 48 h after treatment, enhancement of toxicity was observed by combining Cry4Ba toxin with *X*. *nematophila* (10^8^ CFU) or *P*. *luminescens* (10^8^ CFU), which showed 95% and 85% of larval mortality respectively [10]. In addition, cell suspensions of *Xenorhabdus nematophila* together with *B*. *thuringiensis* subsp. *israelensis* strongly promote the mortality rate of *Ae*. *aegypti* and *Culex pipiens pallens* [11]. Recently, the fluid cultured forms of *X*. *nematophila* and *P*. *luminescens* rapidly caused death of *Ae*. *aegypti* within a few hours and caused delays in the development of pupa and adults [12]. This suggests that *Xenorhabdus* and *Photorhabdus* bacteria are the natural resources for searching for bioactive compounds. However, species identifications of EPNs and their symbiotic bacteria are important fields of research for studies in agricultural and medical fields.

At present, EPNs have been identified in several geographical areas with approximately 100 species of *Steinernema* and 26 species of *Heterorhabditis* [13–27]. However, the diversity and application of both EPNs and their symbionts have not been thoroughly studied in several countries, including Thailand. In our country, six species of *Steinernema* have been found in several provinces: *S*. *siamkayi*, *S*. *websteri*, *S*. *minutum*, *S*. *khoisanae*, *S*. *scarabaei* and *S*. *kushidai*. Additionally, 6 species of *Heterorhabditis*, including *H*. *indica*, *H*. *baujardi*, *H*. *bacteiophora*, *H*. *somsookae*, *H*. *gerrardi* and *H*. *zealandica*, were recorded in Thailand [15, 28–33]. Symbiotic bacteria have been discovered worldwide, with approximately 24 species of *Xenorhabdus* and 5 species of *Photorhabdus*. In Thailand, three species of *Xenorhabdus* were reported, including *X*. *stockiae*, *X*. *miraniensis* and *X*. *japonica*. Additionally, 3 species with 6 subspecies of *Photorhabdus*, including *P*. *luminescens* subsp. *akhurstii*, *P*. *luminescens* subsp. *hainanensis*, *P*. *luminescens* subsp. *laumondii*, *P*. *asymbiotica* subsp. *australis*, *P*. *luminescens* subsp. *namnaonensis* and *P*. *temperata* subsp. *temperata*, were also recorded across the country [15, 33–36].

Most surveyed locations for EPNs and their associated microbes in Thailand were roadside, with minor amounts surveyed from fruit crops, rice fields and river banks [15, 31, 32]. The first novel species of EPN in Thailand, *S*. *siamkayi*, was isolated from soil samples in Phetchabun province [28]. Subsequently, a novel subspecies of symbiotic bacteria, *P*. *luminescens* subsp. *namnaonensis*, was isolated from *H*. *baujardi* from the Nam Nao district in Phetchabun province [36]. This suggests that EPNs and their symbionts may be diverse in Phetchabun province, a mountainous region of central Thailand. Therefore, other areas in Phetchabun province should be explored for EPNs and their symbiotic bacteria. Nam Nao National Park includes the mountainous forests of Phetchabun and Chaiyaphum provinces in Thailand. The flora consists mainly of dry dipterocarps, mixed deciduous, hill evergreens, vast bamboo groves, pine forests and some grassland areas. Despite the interesting location for the surveying of EPNs and their symbiotic bacteria, there is only one report of these organisms in the National Park (Mae Wong) of Thailand. This area is abundant with insect hosts and might lead to the recovery of several species of EPNs and symbiotic bacteria. In addition, four organisms were newly recorded in Mae Wong National Park, namely *H*. *zealandica*, *S*. *kushidai*, *X*. *japonica* and *P*. *temparata* subsp. *temperata*. Therefore, the objectives of this study were to isolate and identify EPNs and their associated *Xenorhabdus* and *Photorhabdus* from Nam Nao National Park of Thailand and to determine the larvicidal activity of these bacteria against *Ae*. *aegypti* and *Ae*. *albopictus*.

## Materials and methods

### Collection of soil samples

The methods for soil collection in Nam Nao National Park of Phetchabun province were approved and permitted by the Department of National Park, Wildlife and Plant Conservation, Thailand (Permission number 0907.4/8245). Overall, 940 soil samples were collected from 188 soil sites in Nam Nao National Park between February 2014 and July 2016. At each site, five soil samples (300–500 g) were collected using a hand shovel. The soil collection process was performed according to Thanwisai et al. [15]. The latitude and altitude of each soil site were determined using a GPS navigator (Garmin nüvi 1250, Taiwan). The moisture and pH of each soil sample were recorded using the soil pH and moisture meter Takemura DM15 (Takemura Electronic Works, Chiba, Japan). The soil temperature of each sample was also measured using a soil survey instrument (Yancheng Kecheng Optoelectronic Technology, Jiangsu, China). Soil texture was also noted. All soil samples were transported in ambient temperature to the Department of Microbiology and Parasitology, Faculty of Medical Science, Naresuan University, Phitsanulok province, Thailand for the isolation of EPNs. Soil parameters (soil temperature, pH and moisture) of samples with and without EPNs were compared statistically using the Mann-Whitney test in the SPSS Program, Version 17 (*P* < 0.05).

### Identification of entomopathogenic nematodes

A baiting technique [37] using *Galleria mellonella* larvae followed by a White trap [38] was performed to isolate EPNs from soil samples. The infective juvenile stages (IJs) of EPNs emerged from the *G*. *mellonella* cadaver and moved to water. The IJs were collected in a culture flask and cleaned by a sedimentation technique, along with several changes of sterile distilled water. The clean IJs were kept in aerated water and stored at 13 °C in a refrigerator. To increase the number and confirm the entomopathogenicity of EPNs, a new *G*. *mellonella* larva was infected with IJs.

The genomic DNA of EPNs was extracted from the IJs as described previously [15]. The genomic DNA was dissolved in 20 μl of sterile distilled water and stored at –20 °C for future use.

A PCR-based analysis and DNA sequencing were conducted to identify *Steinernama* and *Heterorhabditis* species. The primers used for amplifying *Steinernama* 28S rDNA were 539_F (5’GGATTTCCTTAGTAACTGCGAGTG-3’) and 535_R (5’–TAGTCTTTCGCCCCTATACCCTT-3’) [39]. The PCR conditions consisted of pre-denaturation at 95 °C for 1 min, followed by 35 cycles of denaturation at 94 °C for 1 min, annealing at 55 °C for 30 s, extension at 72 °C for 45 s, and a final extension at 72 °C for 7 min. The size of amplification fragment was 870 bp.

The primers used for amplifying *Heterorhabditis* internal transcribe spacer (ITS) were18S_F (5’-TGATTACGTCCCTGCCCTTT-3’) and 26S_R (5’-TTTCACTCGCCGTTACTAAGG-3’) or TW81_F (5’-GTTTCCGTAGGTGAACCTGC-3’) and AB28_R (5’-ATATGCTTAAGTTCAGCGGGT-3’) [40]. The PCR conditions for *Heterorhabditis* consisted of predenaturation at 95 °C for 1 min, followed by 35 cycles of denaturation at 94 °C for 30 s, annealing at 50 °C for 1 min, extension at 72 °C for 1 min and a final extension at 72 °C for 7 min. The PCR product was varied between 800–1,100 bp.

A total of 30 μl of the PCR mixture of each *Steinernama* and *Heterorhabditis* consisted of 3 μl of 10x buffer (1x), 4.2 μl of 25 mM MgCl_2_ (3.5 mM), 0.6 μl of 10 mM dNTPs (200 μM), 1.2 μl of 5 μM forward primer (0.8 μM), 1.2 μl of 5 μM reverse primer (0.8 μM), 0.3 μl of 2.5 unit Taq DNA polymerase (0.1 U/ml), 7.5 μl of DNA solution (20–200 ηg) and 12 μl of sterile distilled water. Thermocycling for *Steinernama* and *Heterorhabditis* was performed in the thermal cycler (Applied Biosystems, Pittsburgh, PA, USA). PCR products were verified by electrophoresis on 1.2% TBE-buffered agarose gel. The PCR products were purified using a Gel/PCR DNA Fragment Extraction Kit (Geneaid Biotech Ltd., New Taipei, Taiwan) as recommended by the manufacturer. The DNA sequencing for both directions was analyzed by the Macrogen Inc. service in Korea.

### Isolation and identification of symbiotic bacteria

Symbiotic bacteria were isolated from the hemolymph of dead *G*. *mellonella* larvae, which had been infected with the IJs of EPNs. The dead larvae of *G*. *mellonella* were surface-sterilized by dipping into absolute ethanol for 1 min and placed in a sterile petri dish to dry. Sterile forceps were used to open the 3rd segment from the head of *G*. *mellonella*. A sterile loop was used to touch hemolymph and streaked on nutrient agar supplemented with bromothymol blue and triphenyl-2,3,5-tetrazolium chloride (NBTA). Colonies of *Xenorhabdus* and *Photorhabdus* were blue and green on NBTA after four days of incubation in the dark at room temperature. A single colony of each isolate of symbiotic bacteria was inoculated in 3 ml of Luria-Bertani (LB) broth and incubated with shaking at 180 rpm overnight (approximately 18–24 h). The genomic DNA of symbiotic bacteria was extracted from bacterial pellets using the Blood/Cell DNA Mini Kit (Geneaid Biotech Ltd., New Taipei, Taiwan). The genomic DNA of symbiotic bacteria was stored at –20 °C for further use in PCR.

PCR-based analysis and *recA* gene sequencing was performed to identify *Xenorhabdus* and *Photorhabdus* species. Primers used were recA_F (5’-GCTATTGATGAAAATAAACA-3’) and recA_R (5’–RATTTTRTCWCCRTTRTAGCT-3’) to obtain an 890 pb amplicon [41]. The PCR conditions for *Xenorhabdus* and *Photorhabdus* were based on a previous report by Thanwisai el at. (2012) [15]. A total of 30 μl of the PCR reaction contained 3 μl of 10x buffer (1x), 4.2 μl of 25 mM MgCl_2_ (3.5 mM), 0.6 μl of 10 mM dNTPs (200 μM), 1.2 μl of 5 μM forward primer (0.8 μM), 1.2 μl of 5 μM reverse primer (0.8 μM), 0.3 μl of 2.5 unit Taq DNA polymerase (0.1 U/ml), 3 μl of genomic DNA solution (20–200 ηg) and 16.5 μl of sterile distilled water. PCR was conducted in a thermal cycler (Applied Biosystems, Pittsburgh, PA, USA). The thermal profile was as previously described [15]. PCR products were visualized on a ethidium bromide-stained 1.2% agarose gel and purified using a PCR clean-up Gel Extraction Kit (Macherey-nagel, Düren, Germany). The DNA sequencing for both directions was analyzed by Macrogen Inc. service in Korea.

### Sequencing and phylogenetic analysis

To identify species of EPNs (*Steinernema* and *Heterorhabditis*) and symbiotic bacteria (*Xenorhabdus* and *Photorhabdus*), comparison of the partially edited nucleotide sequences (*recA*, ITS and 28S rDNA) was performed using the BLASTN program from NCBI. A cut-off of ≥ 97% identity was considered for the same species. The nucleotide sequences were aligned using ClustalW, and a phylogenetic tree was constructed using the maximum likelihood method with the MEGA Version 7.0 program [42].

### Bioassay for larvicidal activity

In this study, five isolates of symbiotic bacteria were selected for using in the bioassay. The symbiotic bacteria, including *P*. *luminescens* subsp. *akhurstii* (bNN121.4_TH), *X*. *stockiae* (bNN112.3_TH), *X*. *japonica* (bNN165.4_TH), *X*. *vietnamensis* (bNN167.2_TH) and *X*. *vietnamensis* (bNN167.3_TH), were used for determining the oral toxicity against *Ae*. *aegypti* and *Ae*. *albopictus* larvae.

The eggs of *Ae*. *aegypti* and *Ae*. *albopictus* (laboratory strain) on a filter paper were purchased from the Taxonomy and Reference Museum of the Department of Medical Sciences at the National Institute of Health of Thailand, Ministry of Public Health, Nonthaburi Province, Thailand. *Aedes* eggs were placed into distilled water to allow hatching of first instar larvae, which were fed with minced food pets. The late third and early fourth instar larvae were used in the bioassays.

*Escherichia coli* ATCC^®^ 25922 and distilled water were used as controls for the bioassays. *Escherichia coli* ATCC^®^ 25922 was inoculated in the LB broth and incubated at 37°C for 24 h, while each isolate of *Xenorhabdus* and *Photorhabdus* was inoculated on 5YS medium (5% (w/v) yeast extract, 0.5% (w/v) NaCl, 0.05% (w/v) NH_4_H_2_PO_4_, 0.05% (w/v) K_2_HPO_4_ and 0.02% (w/v) MgSO_4_.7H_2_O) and incubated at room temperature for 48 h. All broth cultures of bacteria were centrifuged at 10,000 x g for 10 min. The supernatant was discarded and the pellets were suspended in sterile distilled water to an OD_600_ of 0.1 with a spectrophotometer (Metertech SP-880, Taiwan).

Suspensions containing *Xenorhabdus* and *Photorhabdus* was evaluated for toxicity against the larvae (third late to early fourth instars) of *Ae*. *aegypti* and *Ae*. *albopictus*. Distilled water and a suspension of *E*. *coli* ATCC^®^ 25922 were used as controls. In each bioassay, 30 larvae of each mosquito species were placed in three wells of a 24-well plate (10 larvae/well). Subsequently, 2 ml (10^7^–10^8^ CFU/ml) from each bacterial suspension was added to each well. This concentration of symbiotic bacteria showed the enhancement of oral toxicity against *Ae*. *aegypti* [10]. The larvae were fed with minced pet food during the experiment. The mortality rate of larvae was monitored after exposure to bacterial suspensions at 24, 48, 72 and 96 h. Each bioassay was tested in three replicates on different dates. The larvae were considered dead when no movement was detected when teased with a fine sterile toothpick. Larval mortality of *Ae*. *aegypti* and *Ae*. *albopictus* after exposure to symbiotic bacteria was analyzed by survival analysis (Kaplan-Meier Estimate) of SPSS Version 17 (*P* < 0.05).

## Results

### Isolation of entomopathogenic nematodes from soil samples

**Fig 1:**
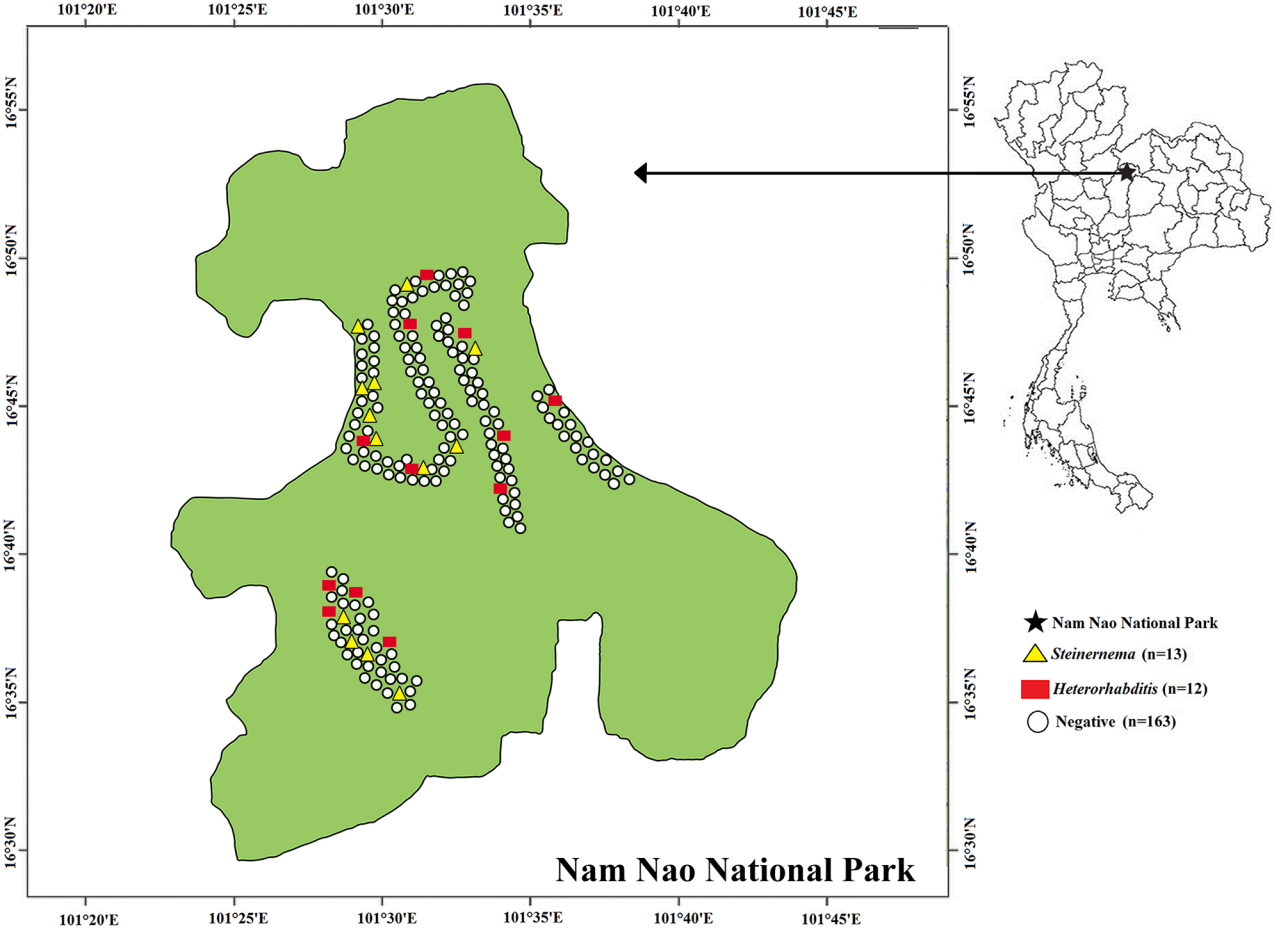
Map of sites for soil collection in Nam Nao National Park, Phetchabun Province, Thailand. A total of 188 soil sampling sites with their statuses of positive or negative for EPNs, *Steinernema* and *Heterorhabditis*, in Nam Nao National Park, Phetchabun Province, Thailand.

Out of the 188 soil sites from Nam Nao National Park, Phetchabun Province, Thailand, 25 tested positive for EPNs (Fig 1). Of the 940 soil samples, 27 (2.87%) were found positive for *Steinernema* or *Heterorhabditis*. This yielded 13 isolates of *Heterorhabditis* and 14 isolates of *Steinernema*. Most EPNs were isolated from loam (96.30%), with the soil pH ranging between 4.8 and 7.0, soil temperature ranging between 19 °C and 30 °C and soil moisture ranging between 1.0 and 8.0 (Table 1). One isolate of the EPNs was isolated from sandy loam and none of the EPNs were isolated from soil with a clay texture.

**Table 1:** Temperature, pH and moisture from soil samples (n = 940) with and without EPNs in Nam Nao National Park, Phetchabun Province, Thailand.

Soil parameter	Soil with EPNs (n = 27)	Soil without EPNs (n = 913)	*P-value* (Mann-Whitney test)
**Temperature (**°C**)**	20–25	19–30	0.055
**pH**	6.2–7.0	4.8–7.0	0.165
**Moisture**	1.0–3.0	1.0–8.0	0.972

### Identification and phylogeny of entomopathogenic nematodes

**Fig 2:**
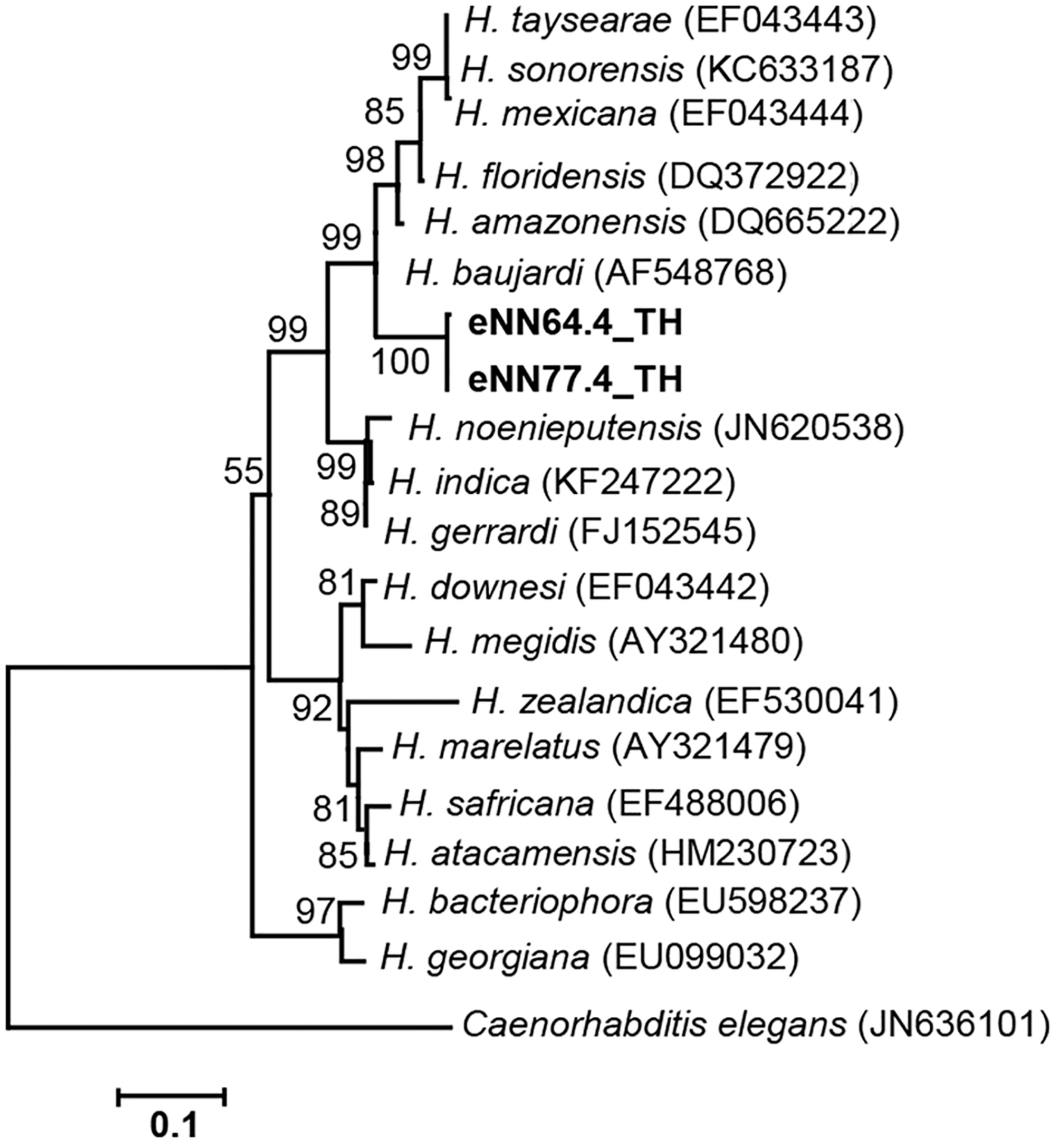
Phylogenetic tree of *Heterorhabditis*. Maximum likelihood tree of a 542 bp of the ITS region from two *Heterorhabditis* isolates from Nam Nao National Park, Phetchabun Province, Thailand (codes ending with TH), together with 18 *Heterorhabditis* sequences downloaded from the GenBank database. *Caenorhabditis elegans* (accession no. JN636101.1) was used as the outgroup. Bootstrap values are based on 1,000 replicates.

**Fig 3:**
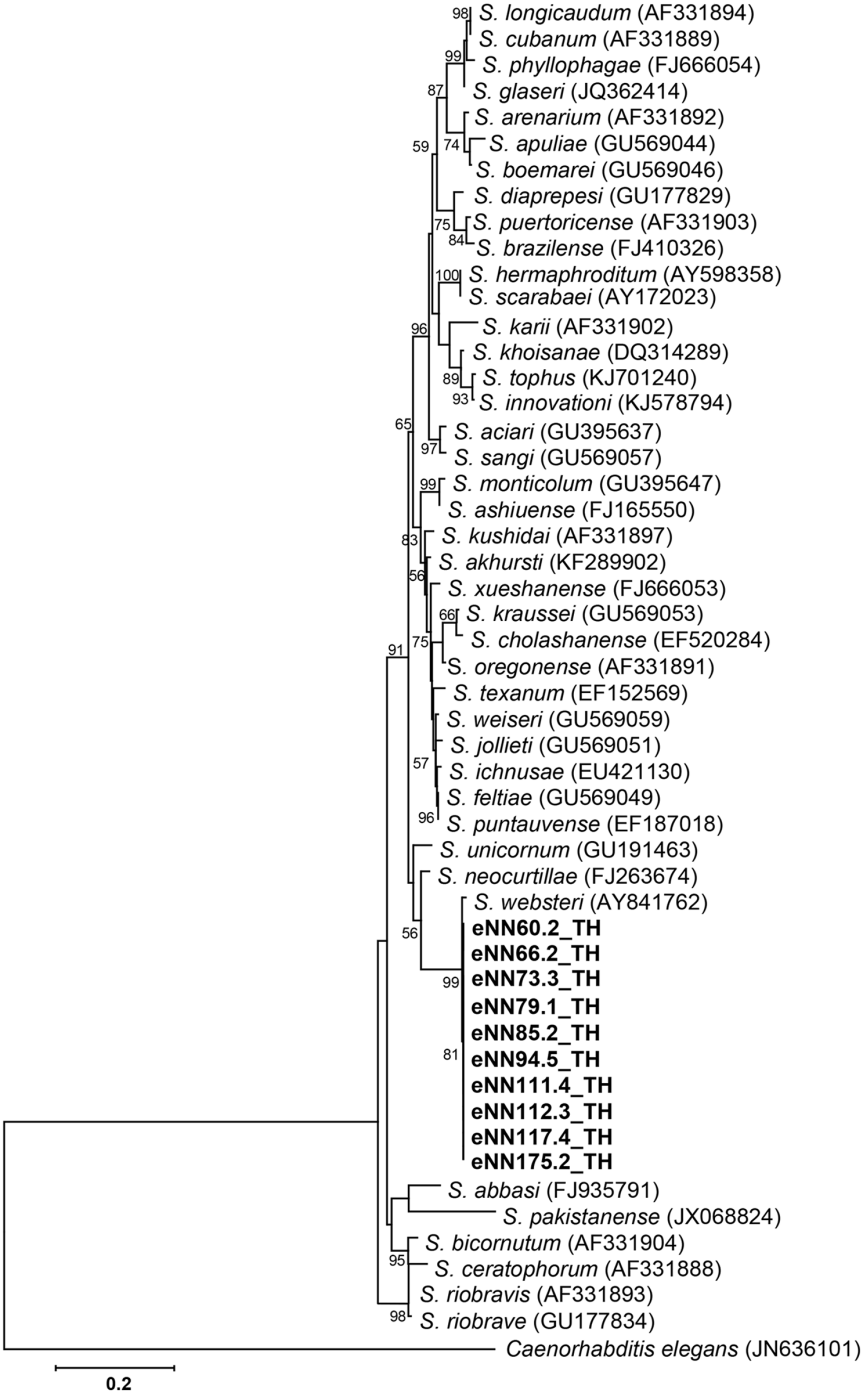
Phylogenetic tree of *Steinernema*. Maximum likelihood tree of a 502 bp of the 28S rDNA region from 10 *Steinernema* isolates from Nam Nao National Park, Phetchabun Province, Thailand (codes ending with TH), together with 41 *Steinernema* sequences downloaded from the GenBank database. *Caenorhabditis elegans* **(**accession no. JN636101.1**)** was used as the outgroup. Bootstrap values are based on 1,000 replicates.

To identify *Heterorhabditis* and *Steinernema* species, PCR-based analysis and sequencing of the ITS and 28S rDNA regions were performed together with a BLASTN search of the edited sequences. Two isolates of *Heterorhabditis* (accession no. MG209260 and MG209261) were identified as *Heterorhabditis baujardi* with 99% identity after BLASTN search using 542 nucleotides of the ITS region. For the genus *Steinernema* (accession no. MG209262, MG209263, MG209264, MG209265, MG209266, MG209267, MG209268, MG209269, MG209270 and MG209271), 10 isolates of *S*. *websteri* were identified with 99% identity after BLASTN search using 502 nucleotides of the 28S rDNA region. *S*. *websteri* was the most recovered species in soil samples from the studied area. The remaining 15 EPN isolates were lost through protozoa and fungal contamination. A phylogenetic tree based on the maximum likelihood method revealed two isolates of the genus *Heterorhabditis* grouped in *H*. *baujardi* (accession no. AF548768.1) (Fig 2) and 10 isolates of the genus *Steinernema* grouped in *S*. *websteri* (accession no. AY841762.1) (Fig 3).

### Isolation, identification and phylogeny of *Xenorhabdus* and *Photorhabdus*

**Fig 4:**
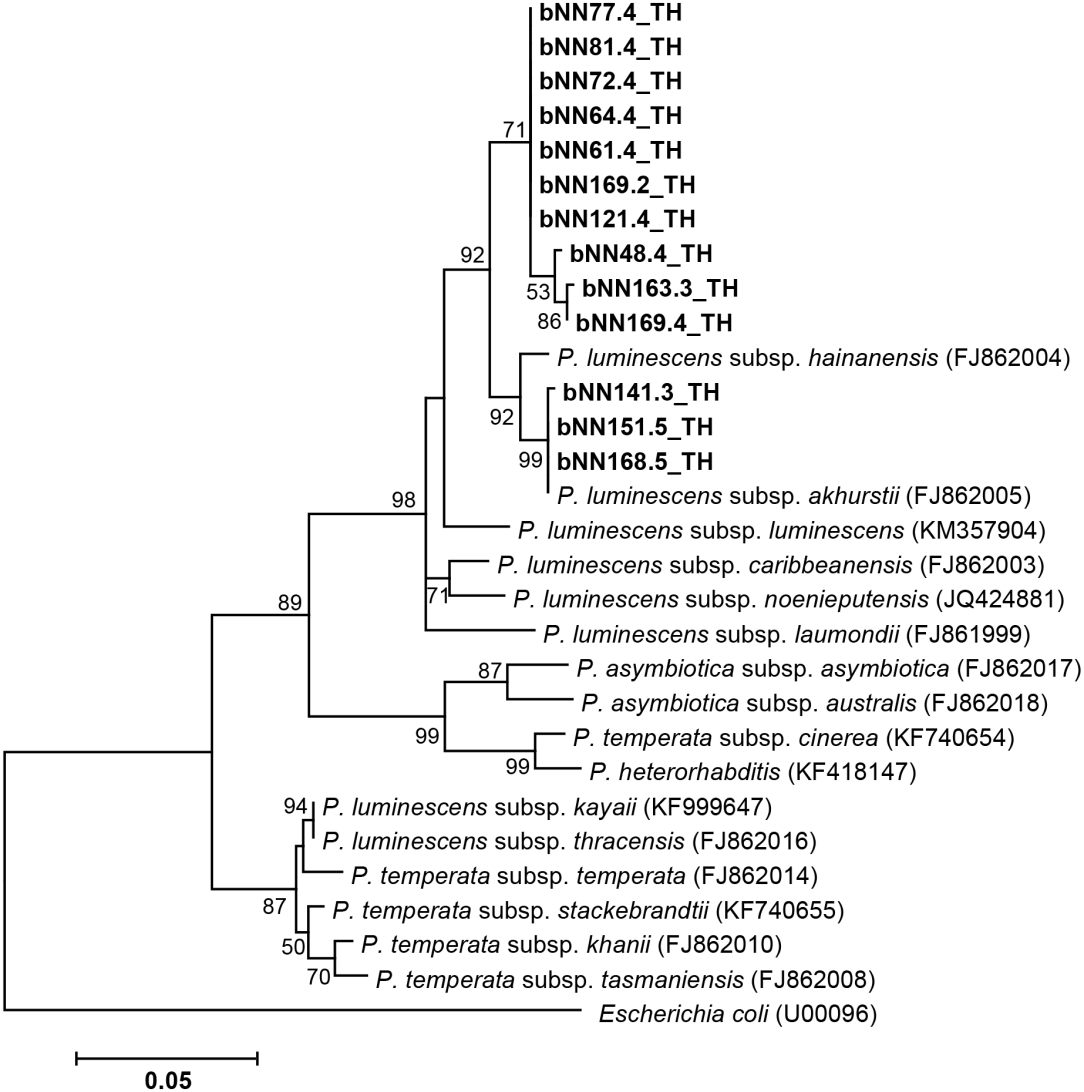
Phylogenetic tree of *Photorhabdus*. Maximum likelihood tree of a 588 bp of the *recA* region for 13 *Photorhabdus* isolates from Nam Nao National Park, Phetchabun Province, Thailand (codes ending with TH), together with 16 *Photorhabdus* sequences downloaded from the GenBank database. *Escherichia coli* (accession no. U00096.3) was used as an outgroup. Bootstrap values are based on 1,000 replicates.

**Fig 5:**
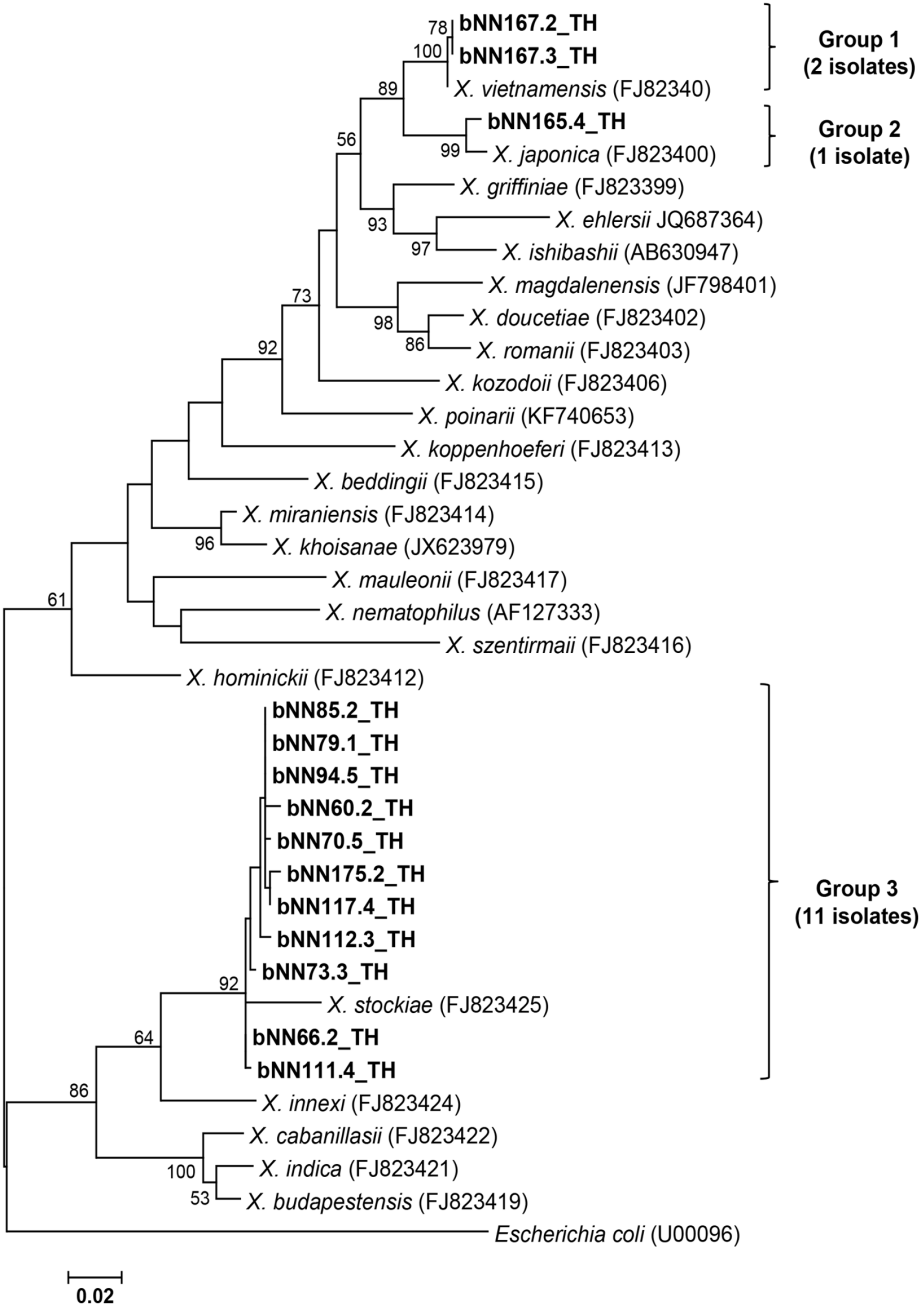
Phylogenetic tree of *Xenorhabdus*. Maximum likelihood tree of a 588 bp of the *recA* gene for 14 *Xenorhabdus* isolates from Nam Nao National Park, Phetchabun Province, Thailand (codes ending with TH), together with 23 *Xenorhabdus* sequences downloaded from the GenBank database. *Escherichia coli* (accession no. U00096.3) was used as an outgroup. Bootstrap values are based on 1,000 replicates.

Based on colony morphology on NBTA agar, 27 isolates of symbiotic bacteria were identified as *Photorhabdus* (13 isolates) and *Xenorhabdus* (14 isolates). A partial sequence of the *recA* gene was amplified and sequenced to identify the *Xenorhabdus* and *Photorhabdus* species. A BLASTN search of the edited sequences was performed to find the identity. A total of 13 isolates of *Photorhabdus* (accession no MG209233, MG209234, MG209235, MG209236, MG209237, MG209238, MG209239, MG209240, MG209241, MG209242, MG209243, MG209244 and MG209245) were identified as *P*. *luminescens* subsp. *akhurstii* with 97–100% identity. Also, 14 isolates of *Xenorhabdus* (accession no MG209246, MG209247, MG209248, MG209249, MG209250, MG209251, MG209252, MG209253, MG209254, MG209255, MG209256, MG209257, MG209258 and MG209259) were identified as *X*. *stockiae* (11 isolates), *X*. *vietnamensis* (2 isolates) and *X*. *japonica* (1 isolate) with an identity ranging from 97–99%. Fig 4 shows the phylogenetic tree based on the maximum likelihood method, which revealed that only one group of *Photorhabdus* (13 isolates in the present study) was closely related to *P*. *luminescens* subsp. *akhurstii* (accession no. FJ862005.1) derived from the NCBI database. Two isolates of *Photorhabdus* (bNN64.4_TH and bNN77.4_TH) were hosted by *H*. *baujardi* (Table 2). In all, 11 isolates of *P*. *luminescens* subsp. *akhurstii* were associated with *Heterorhabditis* sp. (Table 2). Fig 5 shows the phylogenetic tree based on the maximum likelihood method of 14 *Xenorhabdus* isolates in the present study. The group of isolates was divided into 3 groups. Group 1 contained 2 isolates of *Xenorhabdus* (bNN167.3_TH and bNN167.3_TH) and the referenced *X*. *vietnamensis* (accession no. GU481051.1). Group 2 contained only 1 isolate of *Xenorhabdus* (bNN165.4) in the present study and *X*. *japonica* (accession no. FJ823400.1) isolated from *Steinernema kushidai*. Group 3 was composed of 11 isolates of *Xenorhabdus*, which were closely related to *X*. *stockiae* strain TH01 (accession no. FJ823425.1). Most *Xenorhabdus* isolates in the present study were associated with *S*. *websteri* (Table 2). Two *Xenorhabdus vietnamensis* isolates and one isolate of *Xenorhabdus japonica* were hosted by *Steinernema* sp. (Table 2).

**Table 2:** Association between EPN hosts and symbiotic bacteria in Nam in Nam Nao National Park, Phetchabun Province, Thailand.

Association between EPN and their symbiotic bacteria	No. of Associations (isolates)	Code
*Heterorhabditis* associated with *P*. *luminescens* subsp. *akhurstii*	11	NN48.4_TH, NN61.4_TH, NN72.4_TH, NN81.4_TH, NN121.4_TH, NN141.3_TH, NN151.5_TH, NN163.3_TH, NN168.5_TH, NN169.2_TH, NN169.4_TH
*Heterorhabditis baujardi* associated with *P*. *luminescens* subsp. *akhurstii*	2	NN64.4_TH, NN77.4_TH
*Steinernema websteri* associated with *Xenorhabdus stockiae*	10	NN60.2_TH, NN66.2_TH, NN70.5_TH, NN73.3_TH, NN79.1_TH, NN85.2_TH, NN94.5_TH, NN111.4_TH, NN112.3_TH, NN117.4_TH
*Steinernema* associated with *Xenorhabdus stockiae*	1	NN175.2_TH
*Steinernema* associated with *Xenorhabdus vietnamensis*	2	NN167.2_TH, NN167.3_TH
*Steinernema* associated with *Xenorhabdus japonica*	1	NN165.4_TH

### Larvicidal activity of symbiotic bacteria against *Aedes* larvae

**Fig 6:**
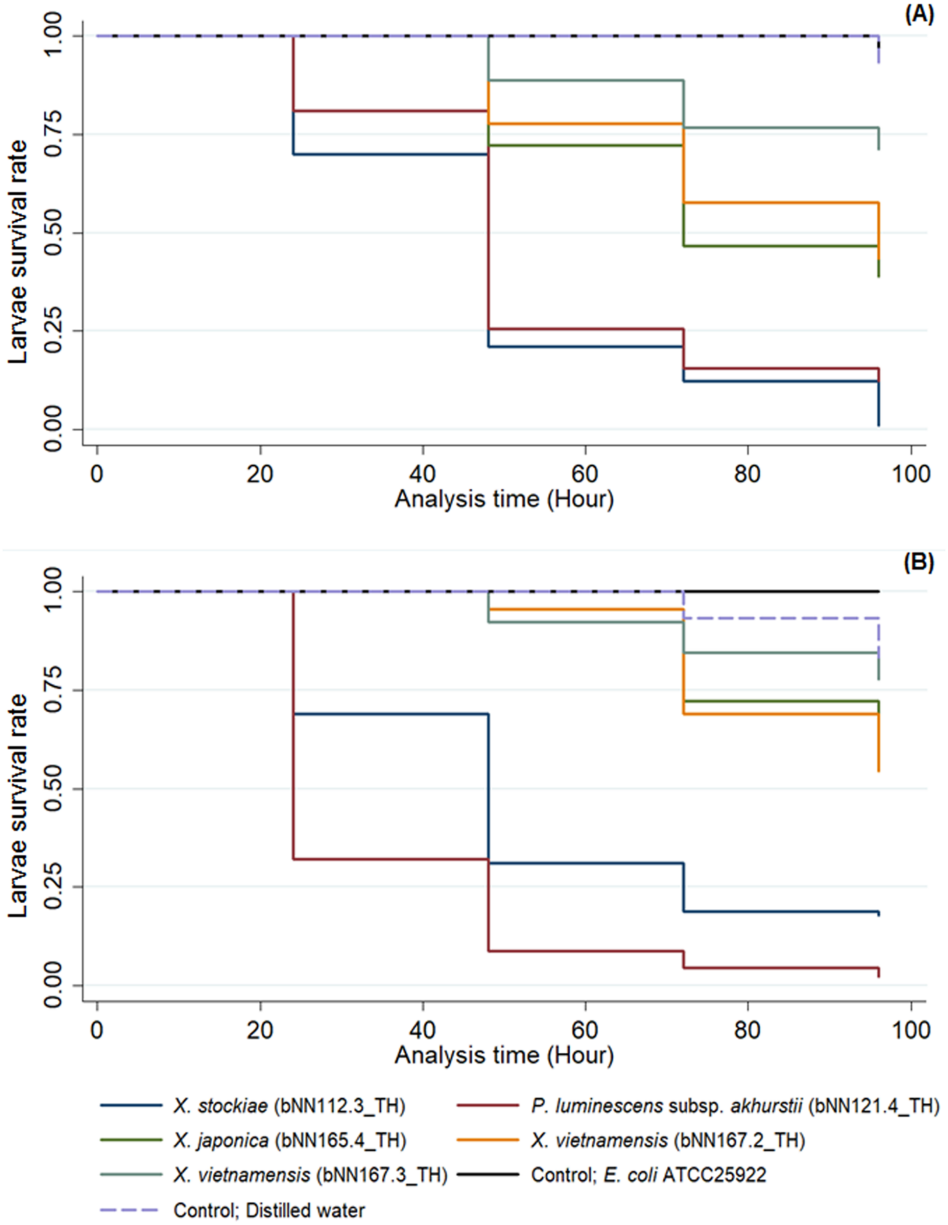
Kaplan-Meier overall survival curves comparing the mortality rate of *Ae*. *aegypti* larvae (A) and *Ae*. *albopictus* larvae (B) after exposure to suspensions of *Xenorhabdus* and *Photorhabdus* bacteria isolated from entomopathogenic nematodes in Nam Nao National Park, Phetchabun Province, Thailand.

The symbiotic bacteria used for the bioassay were selected based on their locations in different groups on the phylogenetic tree. Both, the *Xenorhabdus* and *Photorhabdus* isolates in the present study were assumed to be pathogenic when ingested by *Ae*. *aegypti* and *Ae*. *albopictus* larvae. Fig 6 and Table 3 demonstrate that the highest mortality (99%) of *Ae*. *aegypti* larvae was after exposure to *X*. *stockiae* bNN112.3_TH for 96 h. A significant difference (Kaplan-Meier Estimate, *P-value* = 0.000) was observed when comparing the mortality rate of *A*. *aegypti* larvae between the tested group and the control groups (distilled water and *E*. *coli* ATCC^®^ 25922). The mortality rate in the control groups was low at 0% at 24, 48, and 72 h and 7% for 96 h.

**Table 3:** Mortality rate of *Ae*. *aegypti* and *Ae*. *albopictus* larvae after exposure to suspensions of *Xenorhabdus* and *Photorhabdus* isolated from entomopathogenic nematodes in Nam Nao National Park, Phetchabun Province, Thailand.

Bacteria (Code)	Mortality rate (Mean ± SD)							
	*Ae*. *aegypti*				*Ae*. *albopictus*			
	24 h	48 h	72 h	96 h	24 h	48 h	72 h	96 h
*X*. *stockiae* (bNN112.3_TH)	30 ± 26.03	79 ± 11.71	88 ± 15.40	99 ± 1.92	31 ± 53.89	69 ± 45.26	81 ± 24.11	82 ± 25.02
*P*. *luminescens* subsp. *akhurstii* (bNN121.4_TH)	19 ± 32.72	74 ± 30.97	84 ± 26.94	88 ± 21.17	68 ± 36.63	91 ± 5.09	96 ± 5.09	98 ± 3.85
*X*. *japonica* (bNN165.4_TH)	0 ± 0.00	28 ± 12.62	53 ± 21.86	61 ± 13.47	0 ± 0.00	8 ± 5.09	28 ± 7.70	36 ± 9.62
*X*. *vietnamensis* (bNN167.2_TH)	0 ± 0.00	22 ± 1.92	42 ± 3.84	57 ± 8.82	0 ± 0.00	4 ± 1.92	31 ± 8.39	46 ± 16.78
*X*. *vietnamensis* (bNN167.3_TH)	0 ± 0.00	11 ± 8.39	23 ± 15.28	29 ± 15.03	0 ± 0.00	8 ± 5.09	16 ± 6.94	22 ± 9.62
*E*. *coli* ATCC^®^ 25922	0 ± 0.00	0 ± 0.00	0 ± 0.00	7 ± 0.00	0 ± 0.00	0 ± 0.00	0 ± 0.00	0 ± 0.00
Distilled water	0 ± 0.00	0 ± 0.00	0 ± 0.00	7 ± 0.00	0 ± 0.00	0 ± 0.00	7 ± 0.00	17 ± 0.00

For the bioassay of *Ae*. *albopictus*, the highest mortality of the larvae was 98% after exposure to *P*. *luminescens* subsp. *akhurstii* (bNN121.4_TH) for 96 h, while the control groups (distilled water and *E*. *coli* ATCC^®^ 25922) showed the lowest mortality at 0% at 24 and 48 h, and 7% at 72 h (Fig 6 and Table 3). Moreover, *Ae*. *aegypti* and *Ae*. *albopictus* larvae exhibited a low susceptibility to *X*. *japonica* (bNN165.4_TH) and *X*. *vietnamensis* (bNN167.2_TH and bNN167.3_TH) as indicated by the 22–61% mortality after exposure for 96 h.

## Discussion

Here, we report a low recovery of EPNs (2.87%) from Nam Nao National Park of Thailand. Our findings revealed two genera of EPNs, which consisted of 10 isolates of *Steinernema* and two isolates of *Heterorhabditis*. Most EPNs were isolated from loam, which is consistent with the study by Thanwisai et al. (2012) [15]. In addition, EPNs were not found in clay in the present study.

Soil parameters including temperature, pH, and moisture are important for EPN survival and infectivity. In the present study, the soil moisture was 1.0–3.0% (average 1.8%) for the EPN-positive samples and 1.0–8.0% (average 2.0%) for the EPN-negative samples. This indicated that EPNs, especially *S*. *websteri* and *H*. *indica* (the most common species in Thailand), could live in a restricted range of moisture contents. In addition, soil moisture contents between 4.0–8.0% may not suitable for the survival of EPNs because none were recovered from this range of moisture contents. Although the difference in soil moisture contents found in the present study between the EPN-positive and EPN-negative samples was not significantly great (*P-value* = 0.972), soil moisture is an important factor that supports the ability of EPNs to infect hosts and its survival in different environments [43, 44]. High and low moisture content could affect the movement of EPNs to find a new host; thus, this soil parameter might affect survival rates. The appropriate moisture content for survival is 8–18% for *H*. *indica*, 6–20% for *S*. *themophilum* and 8.0–25.0% for *S*. *glaseri* [45]. This indicates that each species of EPN responds to different levels of moisture. Soil temperatures between EPN-positive samples (20–25 °C) and EPN-negative samples (19–30 °C) in the present study was not significantly great (*P-value* = 0.055). In general, temperatures under 0 °C and above 40°C would lead to EPN death. Temperatures between 10 and 15 °C could limit the movement of EPNs, while temperatures between 30 and 40 °C could result in low infectivity of the EPNs [46]. In the present study, the soil pH between EPN-positive samples (6.2–7.0) and EPN negative samples (4.8–7.0) was not significantly great (*P-value* = 0.165). Soil pH is one of the factors that is important for the infectivity and survival of EPNs. For *Steinernema*, infection and survival rates showed a low level of decrease when the soil pH was between 4 and 8. Rapid decreases in EPN infectivity and survival rates were observed with pH = 10 [47].

Herein, we isolated and identified two isolates of *H*. *baujardi* and 10 isolates of *S*. *websteri*. The most common species found in Nam Nao National Park was *S*. *websteri*, which has previously been isolated from soil samples collected from the roadside areas of several provinces of Thailand [15,31,32]. This EPN was also found in Mae Wong National Park, Kampheang Phet province and central Thailand [33]. This confirmed that *S*. *websteri* was abundant in natural and cultivated fields across the country. *H*. *baujardi*, a less common species that was found in the present study, is also recovered in a low number of isolates across the country [16]. In the early studies of EPNs in Thailand, Tangchitsomkid and Sontirat reported the first isolation of the *Steinernema* and *Heterorhabditis* genera from soil samples from Kanchanaburi, Kalasin, Maha Sarakham, Khon Kaen, Nong Khai, Phichit and Sa Kaeo provinces in Thailand [48]. *Steinernema saimkayi*, a novel species, has been isolated from a soil sample collected from the land used to cultivate tamarind in Phetchabun province [28]. *Steinernema minutum*, a second novel species reported in our country, was recovered from soil samples in Chumphon province, which is in south Thailand [29]. Our finding is consistent with previous studies that the most common species of EPNs in Nam Nao National Park was *S*. *websteri*. However, when comparing our findings with a previous study in Mae Wong National Park, we did not isolate *H*. *zealandica* and *S*. *kushidai*, which may be due to the low distribution of these two EPNs in the National Park.

Of the symbiotic bacteria, all 13 isolates of *Photorhabdus* were molecularly identified as *P*. *luminescens* subsp. *akhurstii*. *Xenorhabdus* species (14 isolates) were molecularly identified as *X*. *stockiae* (11 isolates), *X*. *vietnamensis* (2 isolates) and *X*. *japonica* (1 isolate). In the present study, we found *X*. *stockiae* was associated with *S*. *websteri*, which was consistent with the previous research in Thailand [15, 33]. However, *S*. *websteri* was also symbiotically associated with *X*. *nematophila* [49]. Two isolates of *X*. *vietnamensis* were first identified in Nam Nao National Park of Thailand. Unfortunately, the EPN host *X*. *vietnamensis* was only identified to the genus level in the present study. A previous study in Vietnam reported *X*. *vietnamensis* was associated with *S*. *sangi* [50,51]. The finding of one isolate of *X*. *japocina* in Nam Nao National Park is similar to the finding of Muangpat, who first recorded *X*. *japonica* associated with *S*. *kushidai* in Mae Wong National Park of Thailand [33]. For the genus *Photorhabdus*, *P*. *luminescens* subsp. *akhurstii* was associated with *H*. *baujardi*. Elsewhere, *P*. *luminescens* subsp. *hainanensis*, *P*. *luminescens* subsp. *akhurstii* and *P*. *luminescens* subsp. *laumondii* were associated with *H*. *indica* [15]. The relationship found in this study between *P*. *luminescens* subsp. *akhurstii* and *H*. *baujardi* is a new observation in Thailand.

We demonstrated the toxicity of Thai *Xenorhabdus* and *Photorhabdus* isolates against *Ae*. *aegypti* and *Ae*. *albopictus* larvae. *Xenorhabdus stockiae* bNN112.3_TH and *P*. *luminescens* subsp. *akhurstii* bNN121.4 isolated in the present study have potential to be control agents for *Ae*. *aegypti* and *Ae*. *albopictus*. We suppose that the symbiotic bacteria are pathogenic when ingested by *Aedes* larvae. In general, the diet of *Aedes* larvae are dead and living organic material. These are bacteria, fungi and protozoans that grow on container or are suspended in fluid [52]. These bacteria may produce toxins and secondary metabolites that kill the *Aedes* larvae. To support this scenario, *Xenorhabdus* spp. might produce insecticidal compounds including toxin complexes (Tcs), which play an important role in the pathogenicity of immunosuppression by inhibiting eicosanoid biosynthesis in the target insect [4,53]. *Xenorhabdus nematophila* produces at least eight bacterial metabolites that play crucial roles in suppressing the immune responses of target insects [54]. *X*. *stockiae* PB09 showed miticidal activity against mushroom mites [55]. Several species of *Photorhabdus* have been reported to produce several toxins including toxin complexes (Tcs), make caterpillars floppy (Mcf), *Photorhabdus* insect-related (Pir) proteins and *Photorhabdus* virulence cassettes (PVC) [56]. The Tcs destroy the epithelial cells of the insect midgut and are similar to the δ-endotoxin from *Bacillus thuringiensis* (Bt) [5]. The Mcf is active upon injection and disrupts the insect hemocytes by promoting their apoptosis [57]. The *Photorhabdus Pir* toxin is composed of *Pir*A and *Pir*B, which have been found to be effect against mosquito larvae especially the dengue vectors, *Ae*. *aegypti* and *Ae*. *albopictus* [8, 58]. *Xenorhabdus nematophila* and *P*. *luminescens* showed oral toxicity against *Ae*. *aegypti* [9]. Recently, an enhanced mortality rate of *Ae*. *aegypti* was demonstrated by mixing the Cry4Ba protein of *Bacillus thuringiensis* with *X*. *nematophila* and *P*. *luminescens* [10]. The PVC causes toxicities against a variety of insects such as *Manduca sexta* and *Gallaria mellonella* [59]. Moreover, culture fluids from *X*. *nematophila* caused a delay in pupation and the emergence of adults. The fluids were lethal to larvae, with a cumulative mortality higher than 90% by day 14 [11]. In addition, cell suspensions of *X*. *ehlersii* isolated from *S*. *scarabaei* from Mae Hong Son Province in northern Thailand showed a potential efficacy in killing *Ae*. *aegypti* with 100% mortality [60]. In contrast, *Ae*. *aegypti* and *Ae*. *albopictus* larvae had low mortality rates (4–61%) after exposure to *X*. *japonica* NN165.4_TH, *X*. *vietnamensis* NN167.2_TH and *X*. *vietnamensis* NN167.3_TH. This may have been due to their inability to produce very toxic metabolites to *Aedes*. In addition, different strains of symbiotic bacteria may produce different secondary metabolites. This may give a variable effectiveness in killing the target organisms. Also, the test of the mortality rate of the insects may be related to the variable number of bacterial cells ingested by each larva. It is possible that the symbiotic bacteria are alive in the water and may produce some secondary metabolites in the water. This may be the cause of the larval death.

At present, 2.5 billion people in 100 countries are considered living in areas with a risk infection of the Dengue virus [61,62]. It is transmitted by female mosquitoes mainly of the species *Ae*. *aegypti* and, to a lesser extent, *Ae*. *albopictus*. This mosquito also transmits chikungunya, yellow fever and Zika infection [63,64]. At present, there are no particular treatments or vaccines available to combat infections by these viruses; the only effective method to prevent infection is to avoid mosquito bites [65]. Control measures for *Ae*. *aegypti* and *Ae*. *albopictus* can be achieved using repellents, by cleaning the water source where larvae breed, or applying synthetic insecticides such as organophosphates (temephos and fenthion). The insect growth regulators (diflubenzuron and methoprene) are generally used for the control of mosquito larvae [66–68]. The constant use of such insecticides induces resistance in populations of *Aedes* species [69]. Furthermore, biological control might be an alternative approach to avoid these effects. The advantage of using *Xenorhabdus* and *Photorhabdus* bacteria is that they rapidly kill both *Ae*. *aegypti* and *Ae*. *albopictus* larvae within 48 h. These bacteria also may be non-toxic to humans. *Xenorhabdus* and *Photorhabdus* may thus be effective in all stages of *Aedes* larvae.

## Conclusion

We report a diversity of EPNs and symbiotic bacteria from Nam Nao National Park, Phetchabun Province, Thailand, with the most commonly isolated EPN species being *Steinernema websteri*. The most commonly isolated species of symbiotic bacteria was *X*. *stockiae*. *Xenorhabdus vietnamensis* is the first recorded member of this species of symbiotic bacteria in Thailand. *P*. *luminescens* subsp. *akhurstii* associated with *H*. *baujardi* is a new observation in Thailand. *Xenorhabdus stockiae* and *P*. *luminescens* subsp. *akhurstii* are potential bio-control agents for *Ae*. *aegypti* and *Ae*. *albopictus*. Our findings provide information regarding the diversity of EPNs and symbiotic bacteria in Nam Nao National Park, Phetchabun Province, Thailand and provide a natural resource for further studies of bioactive compounds for controlling mosquitoes. Further investigations of the structure and isolation of the metabolic compounds from *Xenorhabdus* and *Photorhabdus* are needed to update the basic knowledge of the secondary metabolites found in these symbiotic bacteria.
